# The association between common serum adipokines levels and postmenopausal osteoporosis: A meta‐analysis

**DOI:** 10.1111/jcmm.17457

**Published:** 2022-07-05

**Authors:** Linyuan Shu, Yimu Fu, Hui Sun

**Affiliations:** ^1^ Department of Emergency Medicine Shanghai Jiao Tong University Affiliated Sixth People's Hospital Shanghai China; ^2^ Department of Orthopaedic Surgery Shanghai Jiao Tong University Affiliated Sixth People's Hospital Shanghai China

**Keywords:** adipokines, adiponectin, leptin, meta‐analysis, osteoporosis, postmenopausal, resistin

## Abstract

The clinical relevance of blood levels of adipokines in individuals with postmenopausal osteoporosis (PMOP) has not been previously clarified. We performed this meta‐analysis to clarify the association between three common adipokines levels and the occurrence of PMOP. PubMed, Embase, Cochrane library, and China National Knowledgement Infrastructure (CNKI) were searched for collecting articles published before 31 October 2021, without language and status restrictions. Fourteen studies met the selection criteria. Meta‐analysis revealed that blood leptin level was remarkably lower (mean difference [MD], −1.94; 95% confidence interval [CI], −3.83 to −0.06; I^2^ = 96%) and adiponectin level was remarkably higher (MD, 3.48; 95% CI, 2.36 to 4.60; I^2^ = 90%) in individuals with PMOP than healthy individuals with normal bone mineral density (BMD). However, the statistical difference in leptin level was changed after eliminating the confounding influence of leptin sources and assay approaches. Furthermore, a positive association (*r* = 0.28) between leptin level and body mass index (BMI) as well as a negative association (*r* = −0.33) between adiponectin level and BMD was found. Moreover, adiponectin had the highest probability of predicting PMOP (84%). Current evidence suggests that leptin positively affects BMI and adiponectin negatively affects BMD, and adiponectin is the most relevant adipokine negatively associated with PMOP.

## 1 INTRODUCTION

Osteoporosis (OP) refers to an imbalance condition between bone resorption and bone formation, which results in a decrease in bone mineral density (BMD) after increasing bone resorption. Issued data revealed that OP has become a prevalent public health problem worldwide, and its prevalence is estimated to rise from approximately 10 million to more than 14 million by 2020 in the United States.^1^ The diagnosis of OP remains challenging because individuals only experience an asymptomatic condition before it manifests as a low‐trauma fracture of the hip, spine, proximal humerus, pelvis, and/or wrist.^2^ Therefore, more attention should be paid to clarifying the potential mechanisms of OP, which can benefit preventing occurrence or delay progression of OP.

Actually, studies have revealed several factors contributing to the occurrence and progression of OP. Of which, menopause has been especially emphasized because it causes a predominance of bone resorption and therefore, increases the incidence of OP and the risk of bone fractures.^3^ So, postmenopausal individuals will suffer from OP principally because of the detrimental bone turnover caused by oestrogen decline and ageing.^3^ Considering the complexity of regulating physiological and pathological bone biology, other factors were also speculated to regulate the metabolism of bone tissue. Interestingly, epidemiological studies reported a significant positive association between body mass index (BMI) and BMD, and also revealed that reduction in body weight may contribute to bone loss.^4^, ^5^

So far, studies revealed multiple mechanisms that construct the specific association between bone metabolism and changes in fat. Traditionally, fat mass was considered to play an important role in mechanical load or oestrogen secretion as an endocrine organ. However, except oestrogen synthesis in postmenopausal women, adipocytes are also responsible for producing adipokines, such as leptin, adiponectin, and resistin^6^ which act to stimulate inflammatory and anti‐inflammatory responses.^7^, ^8^ It is noted that the synthesis and secretion of adipokines also regulate and modulate energy homeostasis and metabolism.^9^ Moreover, adipokines have also been found to involve in the mediation of bone biology and remodelling.^10^, ^11^

A previous meta‐analysis investigated the influence of blood levels of adipokines on BMD, osteoporotic status, and fracture risk in healthy men and women,^11^ and another Chinese meta‐analysis investigated the difference in adipokines between postmenopausal individuals with OP and healthy individuals with normal BMD^12^; however, the clinical relevance of blood levels of adipokines in postmenopausal individuals with OP has not been definitively clarified in previous meta‐analyses. Therefore, we conducted this meta‐analysis to further investigate the influence of blood levels of three common adipokines, including leptin, adiponectin, and resistin, on the occurrence of OP, BMI, and BMD in postmenopausal individuals.

## 2 MATERIALS AND METHODS

We conducted this meta‐analysis following the Preferred Reporting Items for Systematic Reviews and Meta‐analysis (PRISMA) for Network Meta‐analysis (PRISMA‐NMA)^13^ and the Cochrane methods.^6^ The completed PRISMA checklist has been listed in Table S1. Ethical approval from Institutional Review Board and Informed consent was not applicable to our meta‐analysis.

### 2.1 Search strategy

In 2020, a meta‐analysis investigated the association between adipocytokines and postmenopausal osteoporosis (PMOP), which was published in the Chinese language and identified eligible studies published before February 2020.^12^ We therefore identified potential studies through performed an updated search in PubMed, Embase, the Cochrane library, and China National Knowledgement Infrastructure (CNKI) databases from January 2020 through October 2021, with no language limitations. We used the following key terms to develop the search strategy with Boolean logic operators: leptin, adiponectin, resistin, and PMOP. Additional studies were also manually searched by checking review articles and other relevant material. The type of study was restricted to human studies only. Detailed search strategies are documented in Table S2.

### 2.2 Eligibility and study selection

Two independent authors evaluated and selected relevant studies according to the following criteria: (1) postmenopausal individuals were confirmed as PMOP with recognized criteria in the case group and healthy postmenopausal individuals with normal BMD in the control group; (2) observational studies were considered to be eligible if data of three common adipokines between both groups were available; and (3) studies were published in full‐texts. We also developed the following exclusion criteria: abstracts, studies without data of interest and ineligible design, animal experiments, or the methodological quality were recognized as weak (with a score of <5 in the Newcastle–Ottawa Scale [NOS]). Any disagreements were resolved by consultation with a third senior author.

### 2.3 Data extraction

From the included articles, two independently authors extracted the following information: (1) name of the first author and publication year; (2) country; (3) basic characteristics of individuals including sample size, mean age, mean BMI, and mean time since menopause; (4) details of biochemical examination including sources of indicators and assay approaches; and (5) details of methodology. Moreover, we also extracted correlation coefficients^14^ to further evaluate the relationship between the levels of different adipokines and BMI and BMD. When Spearman's correlation coefficients were reported, we converted it into Pearson's correlation coefficients according to the methods described in previous studies.^15^, ^16^

### 2.4 Quality assessment

We used the NOS to assess the methodological quality of the included studies, which was developed based on a star‐based system.^17^ The methodological quality of an individual study was determined from three aspects as follows: the selection of the study groups, the comparability of the groups, and the ascertainment of the outcome of interest. The total score of >7, 5–6, and <5 was considered high‐, medium‐, and low methodological quality, respectively.

### 2.5 Outcomes of interest

In this meta‐analysis, we defined the difference in leptin, adiponectin, and resistin concentrations between individuals with PMOP and normal individuals as the primary outcomes, and the relationship between the concentrations of adipokines and BMI and BMD as the secondary outcomes.

### 2.6 Statistical analysis

All statistical analyses were conducted using RevMan 5.4 software (Nordic Cochrane Centre, Copenhagen, Denmark). The levels of adipokines were continuous variables, and thus we used the mean difference (MD) with a 95% confidence interval (CI) to express the pooled results because all indicators were calculated using comparable data units. Statistical heterogeneity across studies in individual outcomes was evaluated using Cochrane Q examination^18^ and I^2^ statistic.^19^ Nevertheless, we only used the random‐effects model to perform a meta‐analysis because a relatively definitive conclusion has been achieved about variations between studies in the real world.^20^ Moreover, we first calculated the transformed values of correlation coefficient values using Fisher's transformation method. Then, we performed a meta‐analysis to calculate the summary of Fisher's *Z* values based on a generic inverse‐variance model. Finally, summary *r* values were converted from the summary of Fisher's Z values using recognized formulas. We also tested the robustness of all pooled results using the leave‐one‐out method. It is noted that we calculated standardized levels of adipokines using Z‐scores methods for the purpose of further evaluating the strength of relationships in the different comparisons by introducing the surface under the cumulative ranking curve (SUCRA),^21^ which was estimated using the Bayesian network meta‐analysis with the Aggregate Data Drug Information System version 1.16 (Drug Information Systems, Groningen, The Netherlands). Meanwhile, we also checked publication bias for individual comparison with an accumulated number of more than 10 through generating a funnel plot.^22^ A *p*‐value of <0.05 was considered statistically significant.

### 2.7 GRADE assessment

We used the Grading of Recommendations, Assessment, Development, and Evaluations (GRADE) framework to rate the certainty of evidence. In the GRADE system, the certainty of evidence will be classified as “very low,” “low,” “moderate,” and “high.”^23^ The certainty of evidence begins with a “high” rating for observational studies.^24^ The certainty could be downgraded according to the risk of bias, imprecision, inconsistency, and indirectness; however, the reviewer can upgrade the certainty according to publication bias, large effect, plausible confounding, or dose–response gradient.^25^

## 3 RESULTS

### 3.1 Literature search

**FIGURE 1:**
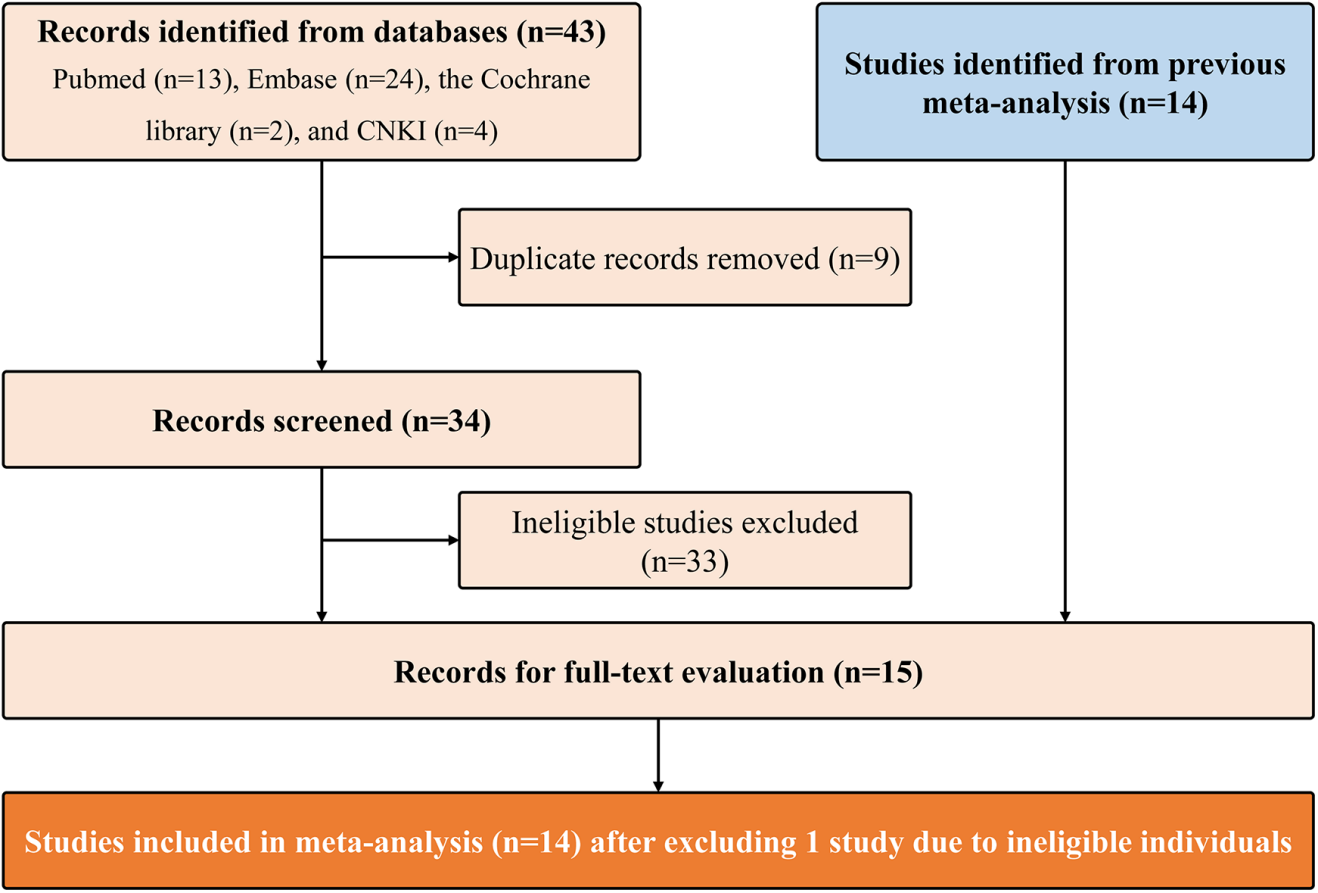
Flow diagram of literature screening. CNKI, China National Knowledgement Infrastructure

We identified 43 records after performing the updated search in the initial retrieval, including Pubmed (*n* = 13), Embase (*n* = 24), the Cochrane library (*n* = 2), and CNKI (*n* = 4). We removed 9 duplicate records through running software, then 33 ineligible studies were excluded after carefully screening the titles and abstracts. A total of 14 studies were identified from a previous meta‐analysis which was published in the Chinese language, and thus, a total of 15 studies were retained for further eligibility assessment. After screening full texts, 14 studies^26^, ^27^, ^28^, ^29^, ^30^, ^31^, ^32^, ^33^, ^34^, ^35^, ^36^, ^37^, ^38^, ^39^ were included in the meta‐analysis. The process of the literature retrieval and screening is shown in Figure 1.

### 3.2 Study characteristics

A total of 14 studies were included in this meta‐analysis finally, which enrolled 615 individuals with PMOP and 499 healthy patients with normal BMD. Of these studies, 8 studies^30^, ^31^, ^33^, ^34^, ^35^, ^37^, ^38^, ^39^ were published in China, and the remaining studies were in Turkey,^29^, ^32^, ^36^ Italy,^28^ France,^27^ and Iraq.^26^ Eleven studies^26^, ^27^, ^29^, ^31^, ^32^, ^33^, ^34^, ^35^, ^36^, ^37^, ^38^ only reported one of three common adipokines and 3 studies^28^, ^30^, ^39^ reported multiple indicators of adipokines. Three studies measured plasma adipokines levels using enzyme‐linked immunosorbent assay (ELISA)^29^, ^36^ and radioimmunoassay (RIA),^32^ and eleven studies measured serum adipokines levels using ELISA^26^, ^28^, ^30^, ^31^, ^33^, ^35^, ^37^, ^39^ and RIA.^27^, ^34^, ^38^ The basic characteristics of the included studies are shown in Table 1.

**TABLE 1:** Characteristics of the included studies (*n* = 14)

Study	Country	No. of patients		Mean age, years		Mean BMI, kg/m^2^		Mean time since menopause, years		Source	Indicators	Assay approach	*r* ^a^	Score^b^
		PMOP	Control	PMOP	Control	PMOP	Control	PMOP	Control					
Xia, 2011	China	41	39	64.82	65.47	23.69	23.87	15.81	16.59	Serum	ADP	ELlSA	BMD	7
Zhang, 2012	China	35	30	62.48	61.75	n.a.	n.a.	10.47	10.07	Serum	LP	RIA	n.a.	6
Wang, 2011	China	26	30	62.08	60.91	25.27	24.5	10.66	9.95	Serum	LP	RIA	n.a.	7
Lv, 2006	China	32	30	59.61	56.2	26.66	24.46	10.09	n.a.	Serum	LP	ELlSA	n.a.	6
Zhang, 2008	China	30	30	61.3	56.8	26.16	25.82	n.a.	n.a.	Serum	LP	ELlSA	BMD	5
Wang, 2015	China	112	60	66.3	65.7	23.5		10.7		Serum	ADP	ELlSA	BMD	8
Zuo, 016	China	58	30	68.9	58.9	22.2	24.8	20	10	Serum	ADP, RT	ELlSA	n.a.	6
Liu, 2010	China	60	56	57.2	5.5	23.2		n.a.		Serum	LP, RT	ELlSA	n.a.	8
Cervellati, 2016	Italy	43	31	58.5	55.3	23.6	26	7.5	3.3	Serum	LP, ADP, RT	ELlSA	BMD	6
Breuil, 2011	France	20	16	73.25	67.3	n.a.	n.a.	12.1	12.9	Serum	LP	RIA	BMI, BMD	5
Odabasi, 2000	Turkey	50	50	61.18	58.3	28.91	29.46	14	12	Plasma	LP	RIA	BMI, BMD	7
Yilmazi, 2005	Turkey	36	30	54.52	54.25	29.33	29.25	9.1	9.02	Plasma	LP	ELlSA	BMI, BMD	7
Kocyigit, 2013	Turkey	42	37	58.2	59.2	28.6	30	12.1	11.6	Plasma	LP	ELlSA	BMI, BMD	7
Al‐Osami, 2018	Iraq	30	30	62.33	60.18	26.45	26.86	15.3	16.3	Serum	ADP	ELlSA	BMI, BMD	5

Abbreviations: ADP, adiponectin; BMI, body mass index; ELISA, enzyme ‐linked immunosorbent assay; LP, leptin; n.a., not applicable; PMOP, postmenopausal osteoporosis; RIA, radioimmunoassay; RT, resistin.

^a^ Pearson's or Spearman's correlation coefficient.

^b^ The methodological quality was determined using the Newcastle–Ottawa Scale (NOS).

### 3.3 Methodological quality

The NOS was used to assess the methodological quality of the included articles. Three studies scored 5, four studies scored 6, five studies scored 7, and the remaining two studies scored 8. In general, the overall methodological quality of included studies was at a moderate level.

### 3.4 Meta‐analysis of adipokines levels in all patients with PMOP

**FIGURE 2:**
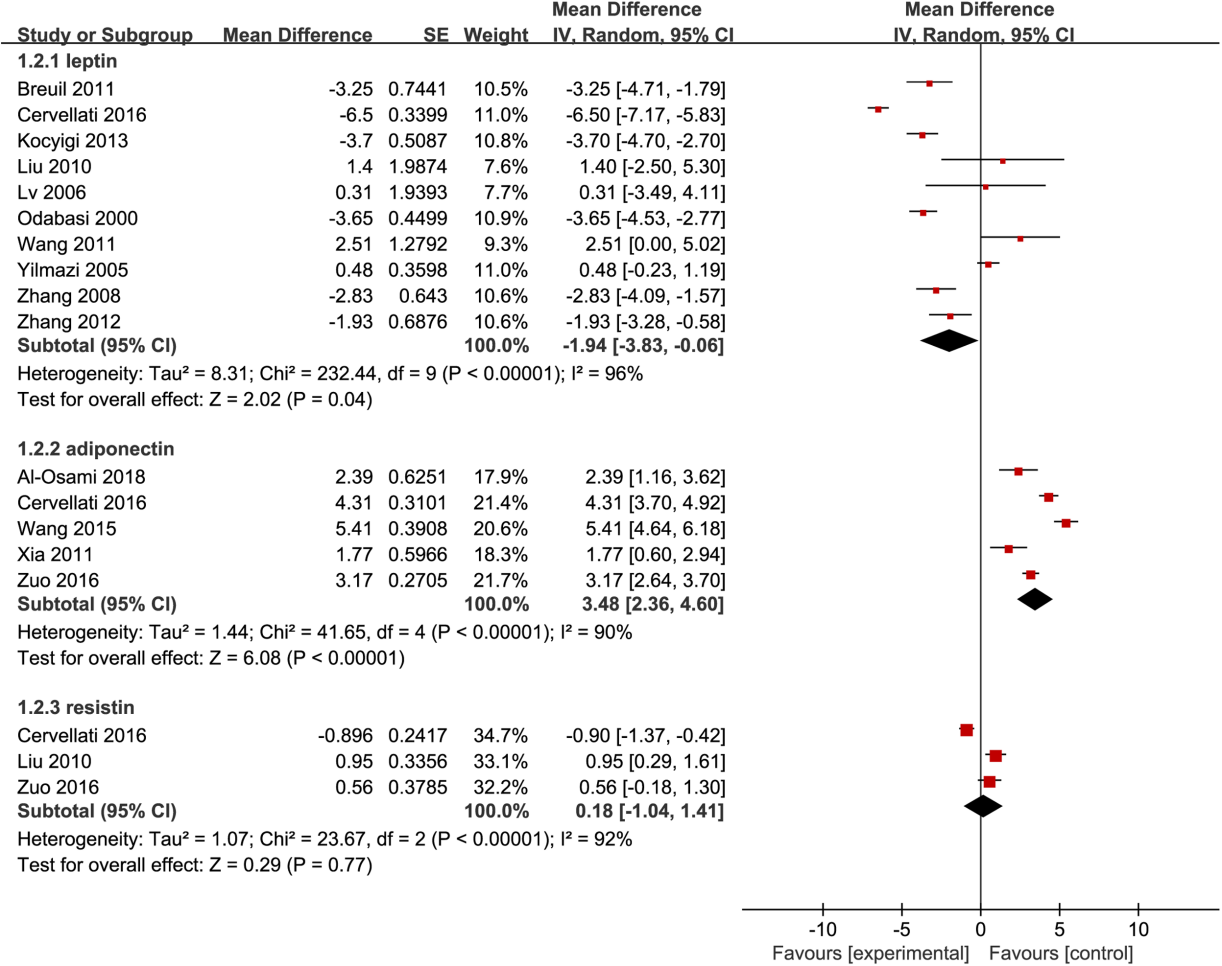
Forest plot of meta‐analysis of differences in blood adipocytokines levels between postmenopausal individuals with osteoporosis and healthy individuals with normal bone mineral density

Among 14 studies included, 10 reported leptin levels in 374 individuals with PMOP and 340 controls. The pooled result suggested that individuals with PMOP had significantly lower leptin levels compared to controls (MD = −1.94 ng/mL, 95% CI, −3.83 to −0.06, *p* = 0.04; Figure 2). Substantial statistical heterogeneity was detected (I^2^ = 96%, *p* < 0.001), we therefore conducted a series of subgroup analyses based on leptin sources and assay approaches to explore potential influencing factors. Pooled results were reversely changed to have no statistical significance although statistical heterogeneity was not obviously decreased, as shown in Figure S1. Moreover, the results of sensitivity analysis also suggested a significant change after omitting one study at one time (Table S3).

A total of 5 studies reported adiponectin levels in 284 individuals with PMOP and 190 controls. As shown in Figure 2, individuals with PMOP had significantly higher adiponectin levels compared to controls (MD = 3.48 μg/mL, 95% CI, 2.36 to 4.60, *p* < 0.001). Although substantial statistical heterogeneity was detected (I^2^ = 90%, *p* < 0.001), no subgroup analysis was conducted because serum adiponectin levels were measured using the ELISA method in these studies. It is noted that the results of sensitivity analysis confirmed the robustness of the pooled result because it was not significantly changed after omitting one study at one time (Table S3).

Among 14 studies included, 3 reported resistin levels in 161 individuals with PMOP and 117 controls. As shown in Figure 2, adiponectin level between individuals with PMOP and healthy controls with normal BMD was comparable (MD = 0.18 pg/mL, 95% CI, −1.04 to 1.41, *p* = 0.77). No subgroup analysis was conducted because serum adiponectin levels were measured using the ELISA method in these studies. We detected substantial statistical heterogeneity for this indicator (I^2^ = 92%, *p* < 0.001), however, we did not conduct a subgroup analysis because serum resistin levels were measured using the ELISA method in these studies. Unfortunately, the pooled result should be cautiously interpreted because there was statistical changes after omitting one study at one time (Table S3).

### 3.5 Meta‐analysis of the relationship of adipokines levels with the BMD

**FIGURE 3:**
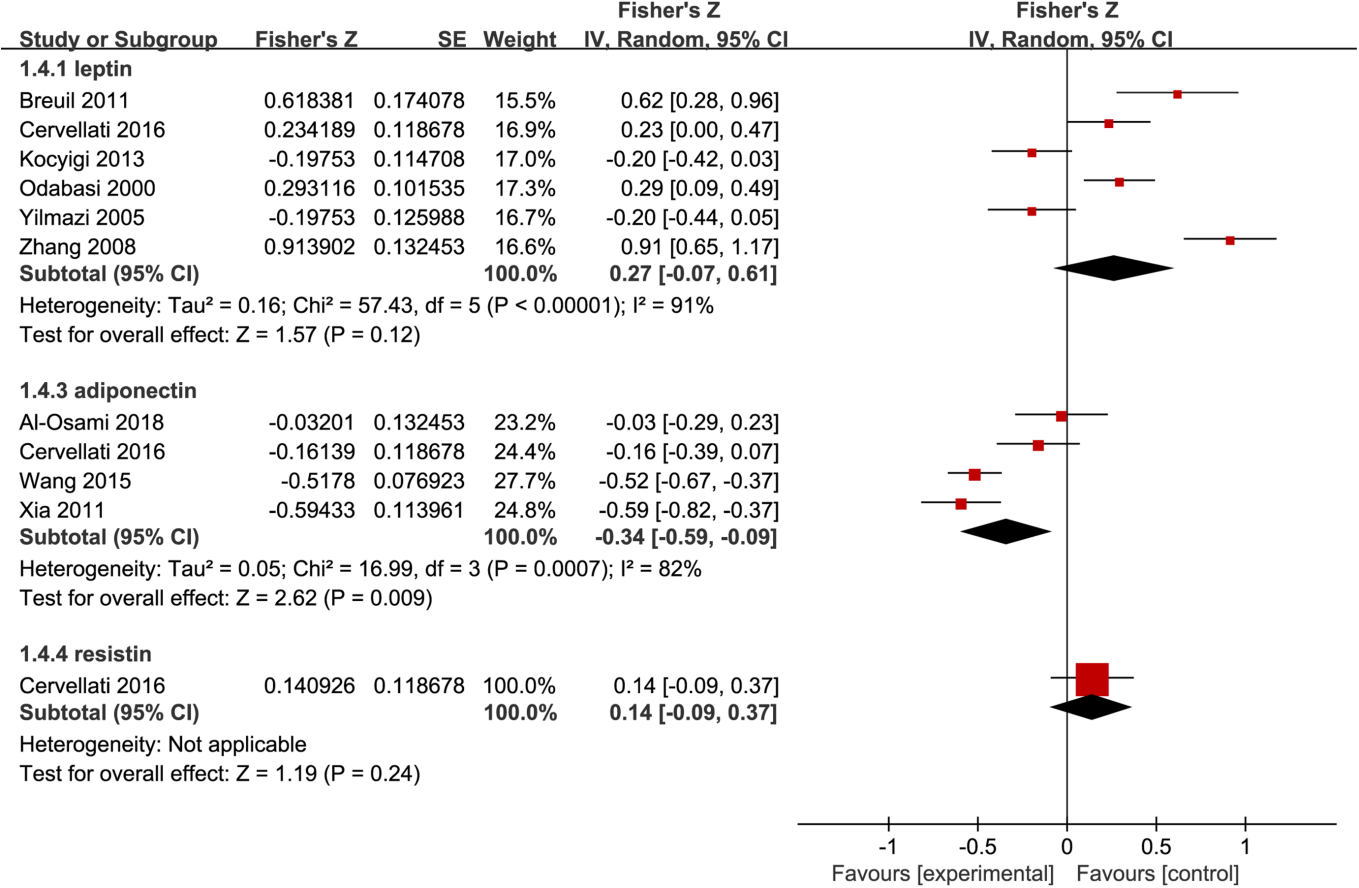
Forest plot of meta‐analysis of the relationship between adipokines and bone mineral density

Among the included studies, 9 studies examined the relationship between adipokines levels and BMD in patients with PMOP and reported the Pearson's or Spearman's correlation coefficients. We performed a pooled analysis of the relationship between adipokines levels and BMD among individuals with PMOP using Fisher's Z transformation. As shown in Figure 3, the meta‐analysis generated a summary Fisher's Z value of 0.27 (95% CI, −0.07 to 0.61, *p* = 0.12), −0.34 (95% CI, −0.59 to −0.09, *p* = 0.009), and 0.14 (95% CI, −0.09 to 0.37, *p* = 0.24) for leptin, adiponectin, and resistin, respectively. Therefore, a summary *r* value was 0.26, −0.33, and 0.14 for leptin, adiponectin, and resistin, respectively. This meta‐analysis of correlation coefficients revealed a significantly negative relationship between adiponectin levels and BMD. However, sensitivity analysis did not confirm the robustness of the pooled results (Table S3).

### 3.6 Meta‐analysis of the relationship of adipokines levels with the BMI

**FIGURE 4:**
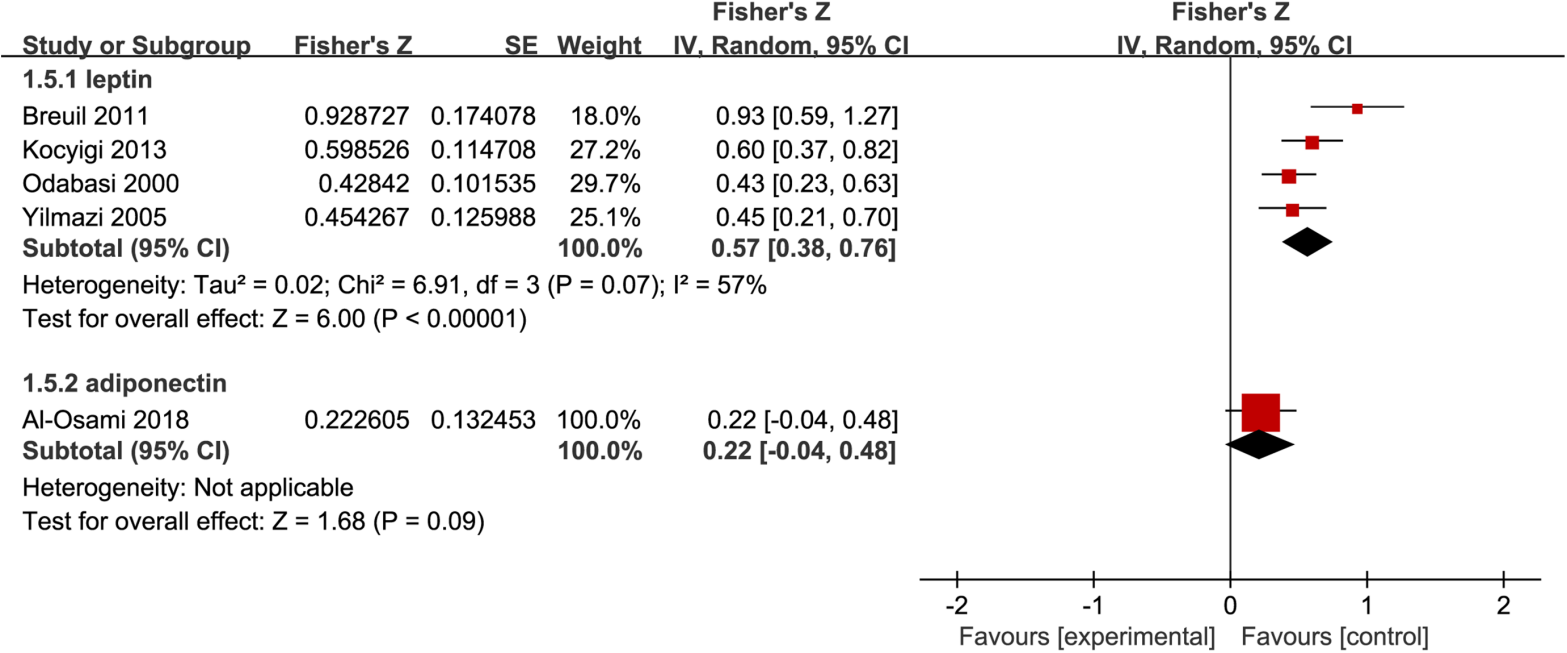
Forest plot of meta‐analysis of the relationship between adipokines and body mass index

Five studies examined the relationship between leptin or adiponectin levels and BMI in patients with PMOP and reported the Pearson's or Spearman's correlation coefficients. We performed a pooled analysis of the relationship between leptin or adiponectin levels and BMI among individuals with PMOP. As shown in Figure 4, the meta‐analysis generated a summary Fisher's Z value of 0.57 (95% CI, 0.38 to 0.76, *p* < 0.001) and 0.22 (95% CI, −0.04 to 0.48, *p* = 0.09) for leptin and adiponectin, respectively. Therefore, a summary *r* value was 0.52 and 0.22 for leptin and adiponectin, respectively. This meta‐analysis of correlation coefficients revealed a significantly positive relationship between leptin levels and BMI, which was further confirmed by the results of sensitivity analysis (Table S3).

### 3.7 Certainty of evidence

The concentration of leptin, adiponectin, and resistin was rated with very low, high, and very low certainty, respectively. Association of leptin, adiponectin, and resistin with BMD was rated with very low, moderate, and very low certainty, respectively. The association of leptin and adiponectin with BMI was rated with moderate and very low certainty, respectively. Details of the certainty of evidence assessment of each outcome were presented in Table **S4**.

### 3.8 Comparative probabilities of the strength of relationships

**FIGURE 5:**
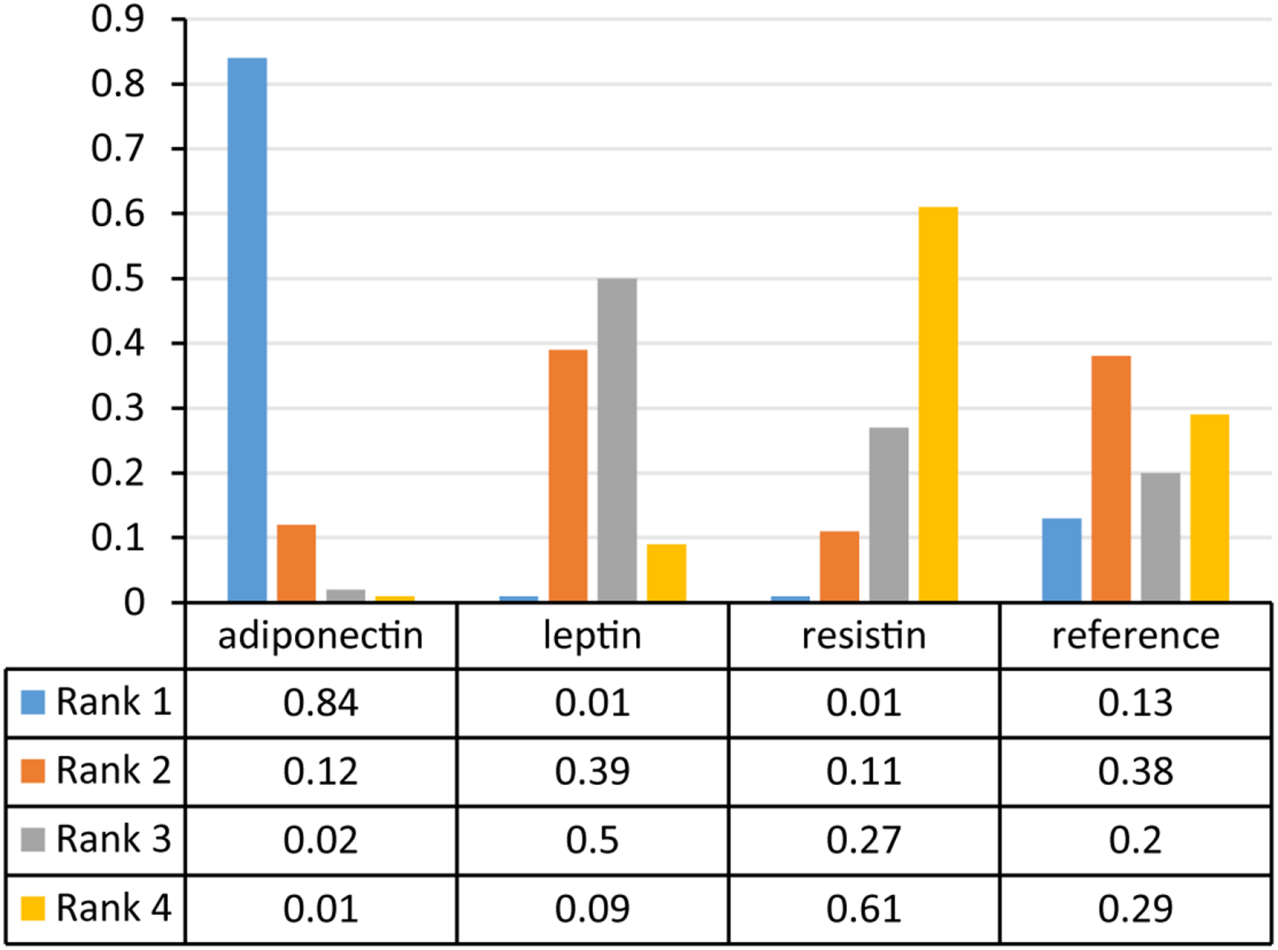
Ranking probability of strength of relationships between adipokines and the occurrence of PMOP. PMOP, postmenopausal osteoporosis

We estimated SUCRA which was calculated following the parameters including 4 chains, 20,000 tuning iterations, 100,000 simulation iterations, the thinning interval of 10, 10,000 inference samples, and a variance scaling factor of 2.5^40^ to rank three available relationships of different adipokines with the occurrence of PMOP. As shown in Figure 5, ranking probability revealed that the relationship between adiponectin levels and the occurrence of PMOP ranked first (84%), followed by the relationship of leptin with PMOP (39%) and the relationship of resistin with PMOP (61%).

### 3.9 Publication bias

A funnel plot was generated to assess the possibility of publication bias among the enrolled studies involving the relationship between leptin levels and the occurrence of PMOP. The symmetrical funnel plot did not identify any publication bias across the studies of patients with PMOP, as shown in Figure S2.

## 4 DISCUSSION

The blood adipokines levels have been found to be associated with BMI and BMD, however, the clinical prevalence of specific adipokines in PMOP remains unclear. We performed this meta‐analysis to further clarify the association between blood levels of three common adipokines including leptin, adiponectin, and resistin, and PMOP through including 14 studies assessing the difference between adipokines and the occurrence of PMOP in postmenopausal individuals. Our meta‐analysis reveals that adiponectin levels are remarkably higher in individuals with PMOP than in healthy individuals with normal BMD. Meanwhile, we also demonstrate a positive relationship between leptin levels and BMI as well as a negative relationship between adiponectin levels and BMD. More, our meta‐analysis also demonstrates that adiponectin is the most relevant adipokine negatively associated with the occurrence of PMOP.

As one of the adipokines, leptin can regulate appetite and weight and is also involved in the proliferation and differentiation of osteoblast^41^, ^42^ and osteoclasts.^41^ Certainly, leptin also affects bone through its actions on the central nervous system, and the role of leptin in regulating bone remodelling has been reported in some clinical epidemiological studies.^11^ In the present meta‐analysis, a positive association between leptin and BMI is demonstrated, however, a definitive association between leptin and BMD is not determined. More importantly, leptin levels in individuals with PMOP are comparable to that in healthy individuals with normal BMD. Many factors may confound the association between leptin levels and BMD, we therefore conducted a subgroup analysis to explore possible confounding factors. Unfortunately, leptin levels are still revealed to be unrelated to the BMD and the occurrence of PMOP. Therefore, more studies are needed to clarify this definitive association.

As a new one of adipokines, adiponectin is exclusively expressed by adipocytes,^7^ and studies reported an inverse relationship between adiponectin levels and visceral fat mass and BMI. Unfortunately, in this meta‐analysis, only one study reported a correlation coefficient between adiponectin levels and BMI and did not reveal a significant association. However, our meta‐analysis demonstrated a significantly negative association between adiponectin levels and BMD, which is consistent with previous findings.^43^, ^44^ More importantly, our meta‐analysis also demonstrated that higher adiponectin concentration is associated with a higher occurrence of PMOP. It is noted that results of SUCRA further revealed that adiponectin may be prevalent adipokine involved in the occurrence of PMOP.

Resistin is mainly expressed by bone marrow and peripheral mononuclear cells (29), and has been speculated to have an association with BMD due to its polymorphisms.^45^ Meanwhile Oh et al. found that, in middle‐aged men, serum resistin levels were inversely associated with lumber BMD.^46^ However, a definitive association between resistin and BMI, BMD, or the occurrence of PMOP has not yet been generated.^11^ In this meta‐analysis, we do not also detect a statistical difference in resistin levels between individuals with PMOP and healthy individuals with normal BMD. Meanwhile, a definitive association between resistin level and BMD is not identified.

It is noted that our meta‐analysis encountered substantial overall statistical heterogeneity for the individual outcome (I^2^ > 90%), and most importantly is that subgroup analysis changed the estimates in terms of the difference in blood leptin levels between postmenopausal individuals with OP and healthy individuals with normal BMD, suggesting that the estimates for leptin should be cautiously interpreted. Meanwhile, heterogeneity of each subgroup remained high after subgroup analyses for available factors, suggesting that several unknown factors contributed to the observed study heterogeneity, such as mean BMI and mean time since menopause.

The present meta‐analysis has several strengths. First, our meta‐analysis firstly investigated the relationships between three common adipokines with the occurrence of PMOP. Second, we introduced the method of calculating SUCRA to determine the relative strength of association of different adipokines with PMOP. Third, we simultaneously estimated the difference of adipokines in levels and the relationships of different adipokines with BMD and BMI. Fourth, although most of the results showed significant heterogeneity, the quality of included studies was at a moderate level.

Despite the novelty of our findings, the following limitations should be further interpreted. First, although we tried our best to control the confounding factors such as adipokines sources and assay approaches, some potential confounding factors such as mean time since menopause may affect the conclusion more or less. Second, since this study lacked effective longitudinal cohort studies, we could not infer the causality of the association between adipokines levels and the occurrence of PMOP. Third, a paucity of studies with extreme sample sizes investigated the association between blood resistin levels and the occurrence of PMOP; thus, the evidence to support it is low. Fourth, we did not register the formal protocol of this meta‐analysis on any public platform, which may introduce bias to the pooled results. However, we strictly followed the methodological framework recommended by the Cochrane handbook to reduce the risk of bias. Fifth, we checked references of topic‐related reviews and included studies to identify the studies missed from the electronic search; however, we did not search other sources for grey literature, which may introduce the risk of missing potentially eligible studies. Sixth, the vast majority of studies originated from Eastern countries; thus, these results should be cautiously extrapolated to Western populations. Seventh, significant heterogeneity was detected for outcomes, which might significantly undermine the validity of the results. However, we conducted a sensitivity analysis to confirm the robustness of some results.

In conclusion, current evidence suggests that blood leptin positively affects BMI and adiponectin negatively affects BMD, as well as adiponectin is the most relevant adipokine negatively associated with the occurrence of PMOP. However, the associations between blood leptin and resistin levels and the occurrence of PMOP, BMI, and BMD should be further verified in future high‐quality studies because most findings were generated from evidence with low or very low certainty in this meta‐analysis.

## AUTHOR CONTRIBUTIONS

**Yimu Fu:** Data curation (equal); formal analysis (equal); writing – original draft (equal). **Linyuan Shu:** Conceptualization (equal); data curation (equal); formal analysis (equal); writing – original draft (equal). **Hui Sun:** Conceptualization (equal); data curation (equal); formal analysis (equal); writing – review and editing (equal).

## CONFLICT OF INTEREST

The authors confirm that there are no conflicts of interest.

## Supporting information


Figure S1

Figure S2

Table S1

Table S2

Table S3

Table S4

## Data Availability

Full datasets generated during and/or analyzed during the current study are available from the corresponding author on reasonable request. Additional data are available in the supplementary materials.
