# Supplementary figures and images for: The association between common serum adipokines levels and postmenopausal osteoporosis: A meta‐analysis
Source: J Cell Mol Med. 2022 Jul 5;26(15):4333–42. doi: 10.1111/jcmm.17457 (PMC9344814; doi:10.1111/jcmm.17457)

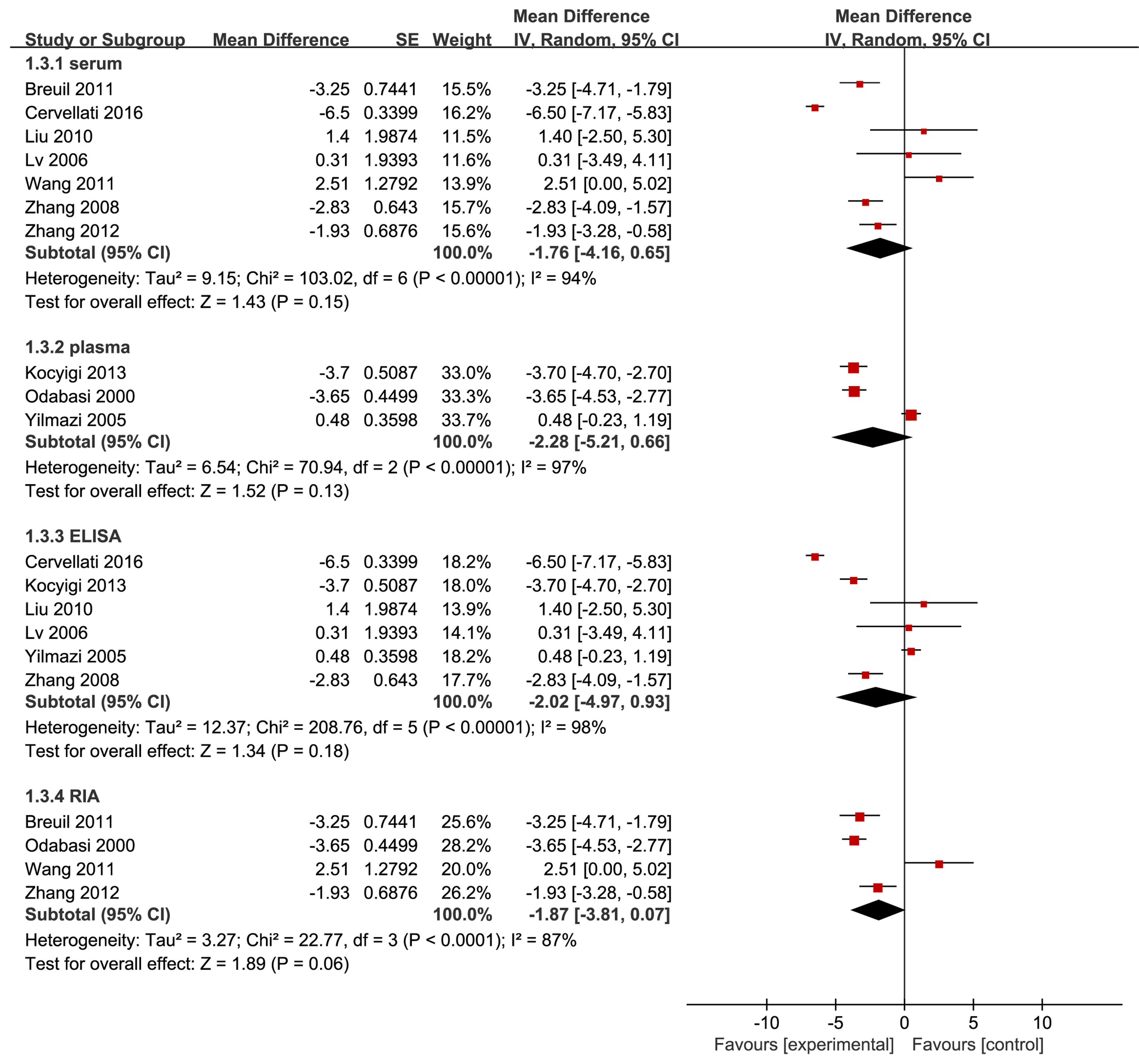

Supplement: Supplementary file 1 — Figure S1 [file JCMM-26-4333-s006.tif]

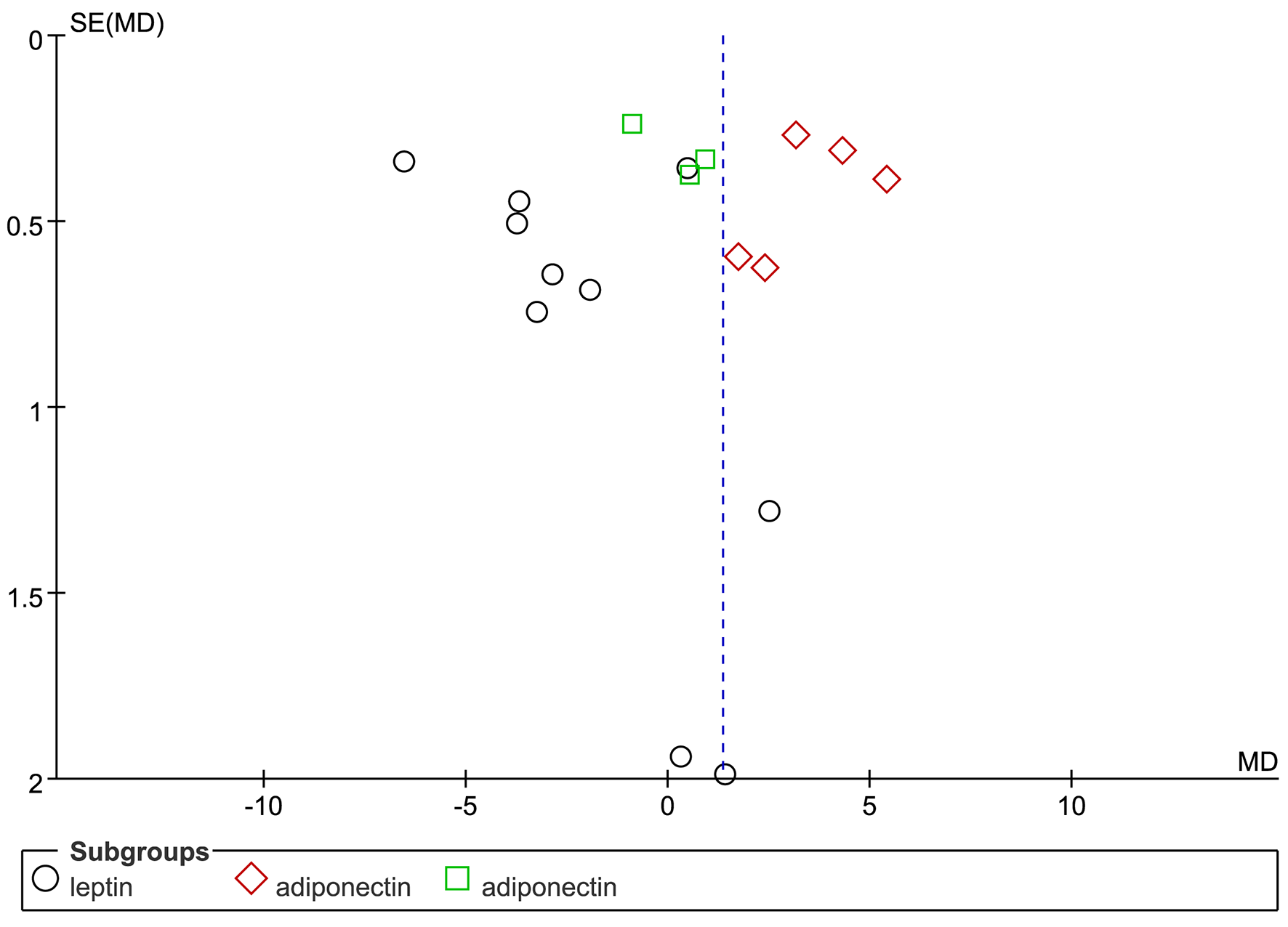

Supplement: Supplementary file 2 — Figure S2 [file JCMM-26-4333-s003.tif]
