# Supplementary material for: The association between common serum adipokines levels and postmenopausal osteoporosis: A meta‐analysis
Source: J Cell Mol Med. 2022 Jul 5;26(15):4333–42. doi: 10.1111/jcmm.17457 (PMC9344814; doi:10.1111/jcmm.17457)
Supplement: Supplementary file 4 — Table S2 [file JCMM-26-4333-s005.docx]

**Table S2.** Search strategy of database.

**Pubmed**

| NO. | Query | Filters |
| --- | --- | --- |
| 13 | ((("Osteoporosis, Postmenopausal"[Mesh]) OR (((((((((postmenopausal osteoporosis[Title/Abstract]) OR (Perimenopausal Bone Loss[Title/Abstract])) OR (Postmenopausal Bone Losses[Title/Abstract])) OR (Post-Menopausal Osteoporoses[Title/Abstract])) OR (Post-Menopausal Osteoporosis[Title/Abstract])) OR (Postmenopausal Osteoporosis[Title/Abstract])) OR (Postmenopausal Osteoporoses[Title/Abstract])) OR (Perimenopausal Bone Losses[Title/Abstract])) OR (Postmenopausal Bone Loss[Title/Abstract]))) OR ((((Postmenopausal[Title/Abstract]) OR (Perimenopausal[Title/Abstract])) OR (Post-Menopausal[Title/Abstract])) AND ((((Bone Loss[Title/Abstract]) OR (Bone Losses[Title/Abstract])) OR (Osteoporoses[Title/Abstract])) OR (Osteoporosis[Title/Abstract])))) AND ((((("Adipokines"[Mesh]) OR "Leptin"[Mesh]) OR "Adiponectin"[Mesh]) OR "Resistin"[Mesh]) OR ((((((Adipokine[Title/Abstract]) OR (Adipocytokine[Title/Abstract])) OR (Adipocytokines[Title/Abstract])) OR (Leptin[Title/Abstract])) OR (Adiponectin[Title/Abstract])) OR (Resistin[Title/Abstract]))) | from 2020 - 2021 |
| 12 | ((("Osteoporosis, Postmenopausal"[Mesh]) OR (((((((((postmenopausal osteoporosis[Title/Abstract]) OR (Perimenopausal Bone Loss[Title/Abstract])) OR (Postmenopausal Bone Losses[Title/Abstract])) OR (Post-Menopausal Osteoporoses[Title/Abstract])) OR (Post-Menopausal Osteoporosis[Title/Abstract])) OR (Postmenopausal Osteoporosis[Title/Abstract])) OR (Postmenopausal Osteoporoses[Title/Abstract])) OR (Perimenopausal Bone Losses[Title/Abstract])) OR (Postmenopausal Bone Loss[Title/Abstract]))) OR ((((Postmenopausal[Title/Abstract]) OR (Perimenopausal[Title/Abstract])) OR (Post-Menopausal[Title/Abstract])) AND ((((Bone Loss[Title/Abstract]) OR (Bone Losses[Title/Abstract])) OR (Osteoporoses[Title/Abstract])) OR (Osteoporosis[Title/Abstract])))) AND ((((("Adipokines"[Mesh]) OR "Leptin"[Mesh]) OR "Adiponectin"[Mesh]) OR "Resistin"[Mesh]) OR ((((((Adipokine[Title/Abstract]) OR (Adipocytokine[Title/Abstract])) OR (Adipocytokines[Title/Abstract])) OR (Leptin[Title/Abstract])) OR (Adiponectin[Title/Abstract])) OR (Resistin[Title/Abstract]))) | |
| 11 | (((("Adipokines"[Mesh]) OR "Leptin"[Mesh]) OR "Adiponectin"[Mesh]) OR "Resistin"[Mesh]) OR ((((((Adipokine[Title/Abstract]) OR (Adipocytokine[Title/Abstract])) OR (Adipocytokines[Title/Abstract])) OR (Leptin[Title/Abstract])) OR (Adiponectin[Title/Abstract])) OR (Resistin[Title/Abstract])) | |
| 10 | (((((Adipokine[Title/Abstract]) OR (Adipocytokine[Title/Abstract])) OR (Adipocytokines[Title/Abstract])) OR (Leptin[Title/Abstract])) OR (Adiponectin[Title/Abstract])) OR (Resistin[Title/Abstract]) | |
| 9 | ((("Adipokines"[Mesh]) OR "Leptin"[Mesh]) OR "Adiponectin"[Mesh]) OR "Resistin"[Mesh] | |
| 7 | (("Osteoporosis, Postmenopausal"[Mesh]) OR (((((((((postmenopausal osteoporosis[Title/Abstract]) OR (Perimenopausal Bone Loss[Title/Abstract])) OR (Postmenopausal Bone Losses[Title/Abstract])) OR (Post-Menopausal Osteoporoses[Title/Abstract])) OR (Post-Menopausal Osteoporosis[Title/Abstract])) OR (Postmenopausal Osteoporosis[Title/Abstract])) OR (Postmenopausal Osteoporoses[Title/Abstract])) OR (Perimenopausal Bone Losses[Title/Abstract])) OR (Postmenopausal Bone Loss[Title/Abstract]))) OR ((((Postmenopausal[Title/Abstract]) OR (Perimenopausal[Title/Abstract])) OR (Post-Menopausal[Title/Abstract])) AND ((((Bone Loss[Title/Abstract]) OR (Bone Losses[Title/Abstract])) OR (Osteoporoses[Title/Abstract])) OR (Osteoporosis[Title/Abstract]))) | |
| 6 | (((Postmenopausal[Title/Abstract]) OR (Perimenopausal[Title/Abstract])) OR (Post-Menopausal[Title/Abstract])) AND ((((Bone Loss[Title/Abstract]) OR (Bone Losses[Title/Abstract])) OR (Osteoporoses[Title/Abstract])) OR (Osteoporosis[Title/Abstract])) | |
| 5 | (((Bone Loss[Title/Abstract]) OR (Bone Losses[Title/Abstract])) OR (Osteoporoses[Title/Abstract])) OR (Osteoporosis[Title/Abstract]) | |
| 4 | ((Postmenopausal[Title/Abstract]) OR (Perimenopausal[Title/Abstract])) OR (Post-Menopausal[Title/Abstract]) | |
| 3 | ("Osteoporosis, Postmenopausal"[Mesh]) OR (((((((((postmenopausal osteoporosis[Title/Abstract]) OR (Perimenopausal Bone Loss[Title/Abstract])) OR (Postmenopausal Bone Losses[Title/Abstract])) OR (Post-Menopausal Osteoporoses[Title/Abstract])) OR (Post-Menopausal Osteoporosis[Title/Abstract])) OR (Postmenopausal Osteoporosis[Title/Abstract])) OR (Postmenopausal Osteoporoses[Title/Abstract])) OR (Perimenopausal Bone Losses[Title/Abstract])) OR (Postmenopausal Bone Loss[Title/Abstract])) | |
| 2 | ((((((((postmenopausal osteoporosis[Title/Abstract]) OR (Perimenopausal Bone Loss[Title/Abstract])) OR (Postmenopausal Bone Losses[Title/Abstract])) OR (Post-Menopausal Osteoporoses[Title/Abstract])) OR (Post-Menopausal Osteoporosis[Title/Abstract])) OR (Postmenopausal Osteoporosis[Title/Abstract])) OR (Postmenopausal Osteoporoses[Title/Abstract])) OR (Perimenopausal Bone Losses[Title/Abstract])) OR (Postmenopausal Bone Loss[Title/Abstract]) | |
| 1 | "Osteoporosis, Postmenopausal"[Mesh] | |

**Embase**

.......................................................

No. Query Results Results Date

#13. #11 AND [embase]/lim AND (2020:py OR 2021:py) 24 9 Nov 2021

#12. #11 AND [embase]/lim 248 9 Nov 2021

#11. #7 AND #10 267 9 Nov 2021

#10. #8 OR #9 101,145 9 Nov 2021

#9. 'adipocytokine'/exp OR 'resistin'/exp OR 89,777 9 Nov 2021

'adiponectin'/exp OR 'leptin'/exp

#8. adipokines:ti,ab,kw OR adipocytokine:ti,ab,kw OR 83,952 9 Nov 2021

adipokine:ti,ab,kw OR adipocytokines:ti,ab,kw OR

leptin:ti,ab,kw OR adiponectin:ti,ab,kw OR

resistin:ti,ab,kw

#7. #3 OR #6 31,263 9 Nov 2021

#6. #4 AND #5 25,388 9 Nov 2021

#5. 'bone loss':ti,ab,kw OR 'bone losses':ti,ab,kw OR 149,938 9 Nov 2021

osteoporoses:ti,ab,kw OR osteoporosis:ti,ab,kw

#4. postmenopausal:ti,ab,kw OR 97,118 9 Nov 2021

perimenopausal:ti,ab,kw OR 'post

menopausal':ti,ab,kw

#3. #1 OR #2 17,940 9 Nov 2021

#2. 'postmenopause osteoporosis'/exp 14,985 9 Nov 2021

#1. 'perimenopausal bone loss':ti,ab,kw OR 9,062 9 Nov 2021

'postmenopausal bone losses':ti,ab,kw OR

'post-menopausal osteoporoses':ti,ab,kw OR

'post-menopausal osteoporosis':ti,ab,kw OR

'postmenopausal osteoporosis':ti,ab,kw OR

'postmenopausal osteoporoses':ti,ab,kw OR

'perimenopausal bone losses':ti,ab,kw OR

'postmenopausal bone loss':ti,ab,kw

**Cochrane library**

ID Search Hits

#1 (postmenopausal osteoporosis):ti,ab,kw OR (Perimenopausal Bone Loss):ti,ab,kw OR (Postmenopausal Bone Losses):ti,ab,kw OR (Post-Menopausal Osteoporoses):ti,ab,kw OR (Post-Menopausal Osteoporosis):ti,ab,kw 4974

#2 (Postmenopausal Osteoporosis):ti,ab,kw OR (Postmenopausal Osteoporoses):ti,ab,kw OR (Perimenopausal Bone Losses):ti,ab,kw OR (Postmenopausal Bone Loss):ti,ab,kw 5370

#3 MeSH descriptor: [Osteoporosis, Postmenopausal] explode all trees 2100

#4 #1 or #2 or #3 5425

#5 (Postmenopausal):ti,ab,kw OR (Perimenopausal):ti,ab,kw OR (Post-Menopausal):ti,ab,kw 21988

#6 (Bone Loss):ti,ab,kw OR (Bone Losses):ti,ab,kw OR (Osteoporoses):ti,ab,kw OR (Osteoporosis):ti,ab,kw 18674

#7 #5 and #6 5452

#8 #4 or #7 5452

#9 (Adipokine):ti,ab,kw OR (Adipocytokine):ti,ab,kw OR (Adipocytokines):ti,ab,kw OR (Leptin):ti,ab,kw OR (Adiponectin):ti,ab,kw 6446

#10 (Resistin):ti,ab,kw 552

#11 MeSH descriptor: [Adipokines] explode all trees 1884

#12 MeSH descriptor: [Leptin] explode all trees 1047

#13 MeSH descriptor: [Adiponectin] explode all trees 894

#14 MeSH descriptor: [Resistin] explode all trees 115

#15 #9 or #10 or #11 or #12 or #13 or #14 6616

#16 #8 and #15 21

CNKI

检索范围：总库 （主题：骨质疏松（精确））OR（主题：骨代谢（精确））OR（主题：骨质减少（精确））OR（主题：骨密度（精确）） AND （（主题：脂肪细胞因子（精确））OR（主题：瘦素（精确））OR（主题：脂联素（精确））OR（主题：抵抗素（精确））） AND （（主题：抵抗素（精确））） 主题定制 检索历史
