# Supplementary material for: The association between common serum adipokines levels and postmenopausal osteoporosis: A meta‐analysis
Source: J Cell Mol Med. 2022 Jul 5;26(15):4333–42. doi: 10.1111/jcmm.17457 (PMC9344814; doi:10.1111/jcmm.17457)
Supplement: Supplementary file 5 — Table S3 [file JCMM-26-4333-s002.docx]

**Table S3.** Sensitivity analysis of outcomes.

Table S3(1). Adipokines levels in all patients with PMOP

| Study omitted | Estimate | [95% Conf. Interval] | |
| --- | --- | --- | --- |
| Leptin | | | |
| Breuil 2011 | -1.7744931 | -3.8462157 | 0.2972295 |
| Cervellati 2016 | -1.492799 | -3.0045192 | 0.01892117 |
| Kocyigi 2013 | -1.7009792 | -3.8530934 | 0.45113489 |
| Liu 2010 | -2.21766 | -4.1745791 | -0.26074076 |
| Lv 2006 | -2.1302652 | -4.097332 | -0.16319838 |
| Odabasi 2000 | -1.6987092 | -3.8935835 | 0.49616513 |
| Wang 2011 | -2.4106359 | -4.3406324 | -0.48063937 |
| Yilmazi 2005 | -2.3769445 | -3.9706473 | -0.78324181 |
| Zhang 2008 | -1.8169432 | -3.9153256 | 0.28143921 |
| Zhang 2012 | -1.9282883 | -4.0018811 | 0.14530455 |
| Combined | -1.9430189 | -3.8307851 | -0.05525262 |
| Adiponectin | | | |
| Al-Osami 2018 | 3.7178767 | 2.4711471 | 4.9646068 |
| Cervellati 2016 | 3.2345753 | 1.732034 | 4.7371168 |
| Wang 2015 | 3.0136092 | 1.9972411 | 4.0299768 |
| Xia 2011 | 3.869972 | 2.7343178 | 5.0056262 |
| Zuo 2016 | 3.538738 | 2.0360372 | 5.0414391 |
| Combined | 3.4799977 | 2.357315 | 4.6026804 |
| Resistin | | | |
| Cervellati 2016 | 0.77834523 | 0.28618151 | 1.270509 |
| Liu 2010 | -0.19713579 | -1.6228464 | 1.2285749 |
| Zuo 2016 | 0.0123173 | -1.7965006 | 1.8211352 |
| Combined | 0.18423616 | -1.0425795 | 1.4110518 |

Table S3(2). Relationship of adipokines levels with the BMD

| Study omitted | Estimate | [95% Conf. Interval] | |
| --- | --- | --- | --- |
| Leptin | | | |
| Breuil 2011 | 0.20731106 | -0.16475248 | 0.57937461 |
| Cervellati 2016 | 0.27998701 | -0.13819569 | 0.69816971 |
| Kocyigi 2013 | 0.36634713 | 0.01218553 | 0.72050875 |
| Odabasi 2000 | 0.26829284 | -0.16272816 | 0.69931382 |
| Yilmazi 2005 | 0.36475155 | 0.00320987 | 0.72629321 |
| Zhang 2008 | 0.13818885 | -0.13786897 | 0.41424668 |
| Combined | 0.27089958 | -0.0666027 | 0.60840186 |
| Adiponectin | | | |
| Al-Osami 2018 | -0.43187177 | -0.66745639 | -0.19628714 |
| Cervellati 2016 | -0.39324999 | -0.69205987 | -0.09444014 |
| Wang 2015 | -0.26689044 | -0.60501361 | 0.07123274 |
| Xia 2011 | -0.25061867 | -0.56234753 | 0.06111023 |
| Combined | -0.3372962 | -0.58951201 | -0.0850804 |

Table S3(#). Relationship of adipokines levels with the BMI

| Study omitted | Estimate | [95% Conf. Interval] | |
| --- | --- | --- | --- |
| Leptin | | | |
| Breuil 2011 | 0.49009982 | 0.36251682 | 0.61768281 |
| Kocyigi 2013 | 0.57367468 | 0.30440876 | 0.84294063 |
| Odabasi 2000 | 0.63479453 | 0.39427105 | 0.87531805 |
| Yilmazi 2005 | 0.6200819 | 0.36695021 | 0.87321353 |
| Combined | 0.57099102 | 0.38435334 | 0.7576287 |
