# Supplementary material for: The association between common serum adipokines levels and postmenopausal osteoporosis: A meta‐analysis
Source: J Cell Mol Med. 2022 Jul 5;26(15):4333–42. doi: 10.1111/jcmm.17457 (PMC9344814; doi:10.1111/jcmm.17457)
Supplement: Supplementary file 6 — Table S4 [file JCMM-26-4333-s004.docx]

**Table S4. GRADE assessment for all outcomes evaluated in this meta-analysis**

| **Certainty assessment** | | | | | | | **Relative Effect (95% CI)** | **Certainty** | **Importance** |
| --- | --- | --- | --- | --- | --- | --- | --- | --- | --- |
| **№ of studies** | **Study design** | **Risk of bias** | **Inconsistency** | **Indirectness** | **Imprecision** | **Other considerations** |  |  |  |
| **leptin concentration in patients with PMOP** | | | | | | | | | |
| 10 | observational studies | serious | serious^a^ | not serious | serious^b^ | all plausible residual confounding would reduce the demonstrated effect | **MD -1.94** (-3.83 to -0.06) | ⨁◯◯◯ Very low | CRITICAL |
| **adiponectin concentration in patients with PMOP** | | | | | | | | | |
| 5 | observational studies | serious | not serious | not serious | not serious | very strong association all plausible residual confounding would reduce the demonstrated effect | **MD 3.48** (2.36 to 4.60) | ⨁⨁⨁⨁ High | CRITICAL |
| **resistin concentration in patients with PMOP** | | | | | | | | | |
| 3 | observational studies | serious | serious^a^ | not serious | not serious | all plausible residual confounding would reduce the demonstrated effect | **MD 0.18** (-1.04 to 1.41) | ⨁◯◯◯ Very low | CRITICAL |
| **association of leptin concentration with BMD** | | | | | | | | | |
| 6 | observational studies | serious | serious^a^ | not serious | not serious | all plausible residual confounding would reduce the demonstrated effect | **Fisher's Z 0.27** (-0.07 to 0.61) | ⨁◯◯◯ Very low | CRITICAL |
| **association of adiponectin concentration with BMD** | | | | | | | | | |
| 4 | observational studies | serious | not serious | not serious | not serious | strong association all plausible residual confounding would reduce the demonstrated effect | **Fisher's Z -0.34** (-0.59 to -0.09) | ⨁⨁⨁◯ Moderate | CRITICAL |
| **association of resistin concentration with BMD** | | | | | | | | | |
| 1 | observational studies | serious | not serious | not serious | not serious | publication bias strongly suspected all plausible residual confounding would reduce the demonstrated effect^c^ | **Fisher's Z 0.14** (-0.09 to 0.37) | ⨁◯◯◯ Very low | CRITICAL |
| **association of leptin concentration with BMI** | | | | | | | | | |
| 4 | observational studies | serious | not serious | not serious | not serious | strong association all plausible residual confounding would reduce the demonstrated effect | **Fisher's Z 0.57** (0.38 to 0.76) | ⨁⨁⨁◯ Moderate | CRITICAL |
| **association of adiponectin concentration with BMI** | | | | | | | | | |
| 1 | observational studies | serious | not serious | not serious | not serious | publication bias strongly suspected all plausible residual confounding would reduce the demonstrated effect^c^ | **Fisher's Z 0.22** (-0.04 to 0.48) | ⨁◯◯◯ Very low | CRITICAL |

**CI:** confidence interval

#### Explanations

a. 95% confidence intervals of all studies were not consistent almost.

b. A broad 95% confidence interval was generated.

c. only one study reported this outcome.
